# Poly (Dimethylsiloxane) Coating for Repellency of Polar and Non-Polar Liquids

**DOI:** 10.3390/polym12102423

**Published:** 2020-10-21

**Authors:** Hila Monder, Leo Bielenki, Hanna Dodiuk, Anna Dotan, Samuel Kenig

**Affiliations:** Department of Polymer Materials Engineering, Shenkar College, Ramat Gan 52526, Israel; Hilamonder128@gmail.com (H.M.); Leobielenki@gmail.com (L.B.); Hannad@shenkar.ac.il (H.D.); Adotan@shenkar.ac.il (A.D.)

**Keywords:** contact angle (CA), contact angle hysteresis (CAH), surface tension, dielectric constant, solubility parameter

## Abstract

The wettability of poly (dimethylsiloxane) (PDMS) coating on plasma-treated glass was studied at room temperature using polar and non-polar liquids. The wettability was investigated regarding the liquids’ surface tensions (STs), dielectric constants (DCs) and solubility parameters (SPs). For polar liquids, the contact angle (CA) and contact angle hysteresis (CAH) are controlled by the DCs and non-polar liquids by the liquids’ STs. Solubility parameter difference between the PDMS and the liquids demonstrated that non-polar liquids possessed lower CAH. An empirical model that integrates the interfacial properties of liquid/PDMS has been composed. Accordingly, the difference between the SPs of PDMS and the liquid is the decisive factor affecting CAH, followed by the differences in DCs and STs. Moreover, the interaction between the DCs and the SPs is of importance to minimize CAH. It has been concluded that CAH, and not CA, is the decisive attribute for liquid repellency of PDMS coating.

## 1. Introduction

Super-repellent surfaces capable of repelling both polar and non-polar liquids have stimulated considerable interest due to their importance in various industrial applications such as antifogging, anti-icing, inks, oil/water separation, etc [1,2,3]. Most of the research work related to surface repellency has focused on maximizing static contact angles (CAs). However, static CAs do not always consider the dynamic repellency phenomenon. Inhomogeneity of the surface, such as surface roughness or chemical heterogeneity, forms retaining sites for the droplet at the contact line, hence changing the apparent CAs [4]. In this case, the wetting phenomenon is dynamic, and the CAs should refer to advancing $\left(\theta_{a}\right)$ and receding $\left(\theta_{r}\right)$ contact angles [5,6]. The dynamic angles are usually different from the static Cas, while the advancing CA is the maximum CA value, and the receding CA is the minimum value [7,8,9]. The difference between the advancing and receding CA is the CA hysteresis (CAH), which can be measured by the sessile drop method by which the advancing and receding CA can be obtained when the liquid is added to or withdrawn from a liquid droplet slowly [7,8,9,10]. The measured CAH corresponds to the substrate minimum tilt angle (TA) needed to detach and delink the drop from an inclined surface [10,11].

Most of the research on liquid repellency has been focused on rough superhydrophobic surfaces achieved by mimicking nature, particularly the lotus leaf [1,12,13,14,15]. The lotus leaf is characterized by self-cleaning and water-repellent properties, accomplished by the micro/nano two-scale hierarchical structure [16]. Combining both roughness and extremely low surface energy is critical for obtaining omniphobicity, i.e., repellency of both polar and non-polar liquids. Repelling non-polar liquids is more difficult due to their low surface tension values (<30 mN/m), enabling them to wet almost any surface [3,17,18].

In contrast to rough superhydrophobic coating, only a few studies have been dedicated to repellency of polar and non-polar liquids from smooth (or relatively smooth) surfaces [19,20,21,22,23,24,25,26,27]. Wang and McCarthy [28] studied the attributes of slippery omniphobic covalently attached liquid (SOCAL) coating. By grafting polydimethylsiloxane (PDMS) brushes to a substrate, they created a smooth surface with a liquid-like behavior, which displayed water CAH lower than 5°, and even lower CAH for non-polar liquids such as n-hexadecane (below 2°), regardless of the static CA magnitude. For achieving super-repellent surfaces, the mobility of the tethered chains is a critical factor [21,23,25,29,30]. The chain structure and its chemical functionality are the main parameters affecting the interaction with the probe liquid. It was recognized that enhanced chain mobility allows the sliding of the drop off the surface [31]. It is common knowledge that the static CAs of PDMS based smooth surfaces are dependent on the liquid’s surface tension, but the mechanism of dynamic wettability is less understood. Cheng et al. [29] suggested that the dynamic wettability of PDMS brush coating is influenced by the miscibility of the brushes with the probe liquids. They claimed that when a drop of polar liquid is placed on the surface, a “discrete liquid–liquid interface” is obtained. In contrast, when the surface brushes are in contact with non-polar liquids (alkanes), which are considered good solvents for PDMS, a “blended liquid–liquid interface” is achieved. This unique interfacial phenomenon is characterized by the ability of the PDMS brushes to interact and be swollen by the non-polar liquids, leading to an increase in the brushes’ mobility and consequently enhancing the liquid-like behavior of the PDMS coating. Urata et al. [8] studied the molecular conformation of surface-tethered alkyl groups interacting with a variety of probe liquids and their wettability by measuring the static and dynamic contact angles. Thus, they demonstrated that a good correlation exists between the CAH and the dielectric constants of the probe liquids. When the dielectric constant was lower than 30, the CAH was less than 3° and the TA was less than 5°, regardless of the static CA. Conversely, when the dielectric constant was higher than 34, the CAH increased significantly. In addition, no correlation was found between the dynamic dewettability and other properties like molecular weight/volume, density, and viscosity.

Herein, this study’s objective is to investigate the effect of the liquid/PDMS interfacial properties on the wettability, especially on CAH. The SOCAL coating described by Wang and McCarthy [28] is selected as the model surface. PDMS has low surface tension (~20 mN/m) and is in the rubbery state at room temperature due to its low glass transition temperature (Tg) (−125 ℃) [25,32,33] and large free volume [34]. The large free volume enables the mobility of the PDMS chains attributing liquid-like properties to the solid surface [21]. As pointed out earlier, the liquid’s physical properties, such as surface tension, solubility parameter and dielectric constant, are suggested to affect the CAH with no definite conclusions. Thus, this study consists of a comprehensive analysis of the interfacial wetting dynamics between PDMS coating and a variety of polar and non-polar liquids considering their surface tension, dielectric constant, and solubility parameters.

## 2. Experimental

### 2.1. Materials

Toluene (AR-b), dimethylformamide (DMF) (AR), acetonitrile (AR), ethanol absolute (dehydrated AR-b), 1,4-dioxane (anhydrous), n-hexadecane (99%), 2-propanol (IPA) (HPLC), sulfuric acid (95–98%), dimethyl sulfoxide (DMSO) (AR), 1-propanol (AR), and 1-butanol (AR) were all purchased from BioLab, Israel. Dimethyldimethoxysilane (DMDMS) (95%), diiodomethane (99%), and 1-octanol (99%) were purchased from Sigma-Aldrich, Israel. All materials were used as received without any further purification. Soda-lime glass slides were purchased from SAIL BRAND, Hydrabad, India.

### 2.2. Preparation of PDMS Brush Coating

The PDMS coating was prepared using a procedure described in previous work [28]. Wang and McCarthy studied multiple reaction conditions, various solvents and acid concentrations. They found that the coating presented the lowest CAH was the system with IPA as the solvent and H_2_SO_4_ at a concentration of 1.0 wt%. Consequently, glass surfaces (25 × 25 mm^2^ [2]) were rinsed with IPA and pre-treated with air plasma (electronic diener, using a vacuum system) for 2 min. A solution of 10 g of IPA, 1 g of DMDMS, and 0.1 g of sulfuric acid was prepared in a container, stirred for 30 s and left at room temperature for 30 min. Then, the clean air plasma pre-treated glass slide was submerged in the solution for 10 s. The glass was allowed to dry at room temperature for 20 min, and then was rinsed with water, IPA and toluene (in this order) to remove unreacted components.

### 2.3. Contact Angle Measurements

CAs (static, advancing and receding angles) were measured using a tensiometer (OCA20, DataPhysics, Filderstadt, Germany). All measurements were carried out at room temperature, using the different probe liquids: water, DMSO, DMF, acetonitrile, ethanol, 1-propanol, 1-butanol, 1-octanol, diiodomethane, 1,4-dioxane, toluene and n-hexadecane. Static contact angles were measured by dispensing a 5 μL drop of the probe liquid. Advancing and receding contact angles were recorded, while 5 μL drop of the probe liquid was added to and withdrawn from the surface, respectively. All reported values are an average of 5 measurements, and the error values are in the range of  ±2°.

### 2.4. Atomic Force Microscopy

The surface morphology and smoothness of the coatings were analyzed by atomic force microscopy (AFM) (Bruker Multimode AFM, Santa Barbara, California). The scans were taken in two modes: tapping mode using an AC240 probe from Olympus, and peak force mode (ScanAsyst) using special tips (HPI and PNP-TRS from Bruker). AFM images were analyzed using special software (Gwyddion 2.54 software).

## 3. Results and Discussion

To investigate the effect of the probe liquids’ properties on the wettability of the PDMS-coated substrate, the static CAs and CAHs were measured. The PDMS coating was prepared by condensation reaction ensuring covalent bonding of DMDMS to the plasma-treated glass surface. The chosen probe liquids possess, at room temperature, a variety of properties and cover a wide range of polar and non-polar properties, as listed in Table 1. The surface tensions values ranged from 22.3 to 72.8 mN/m, the dielectric constants values ranged from 2.1 to 79.7, and solubility parameters ranged from 8 to 23.4 ca^0.5^/cm^1.5^.

### 3.1. Wettability of Polar and Non-Polar Liquids

**Figure 1:**
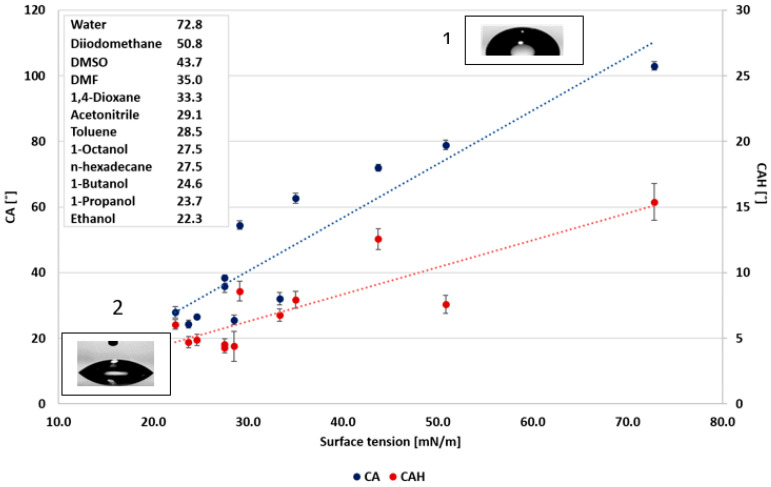
Wettability as a function of surface tension of probe liquids. (**1**). Contact angle (CA) of water (**2**) CA hexadecane.

**Figure 2:**
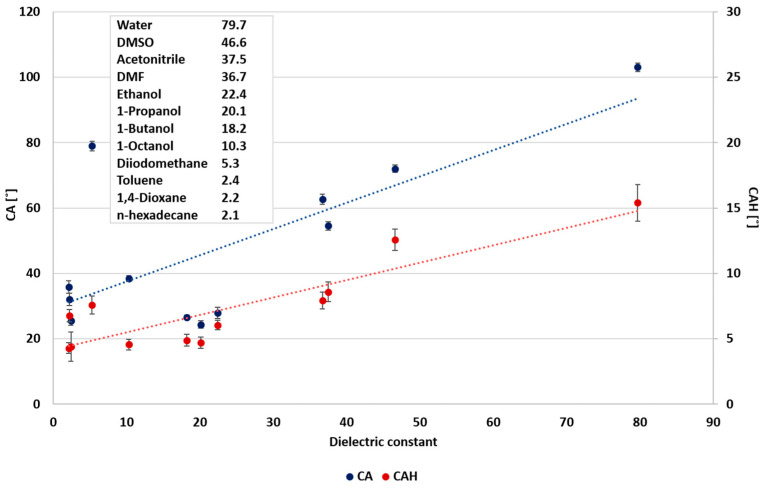
Wettability as a function of the dielectric constant of probe liquids.

In the first stage of the study, the relationships between surface wettability and the liquids’ surface tension and dielectric constant were explored. Urata et al. [8] correlated the static CA and the liquids’ surface tension. Accordingly, the static CA was reduced with the decrease in the liquids’ surface tension. However, they found no correlation between the dynamic CA (CAH) and the surface tension. Nevertheless, they showed that the dynamic CA could be correlated with the dielectric constant of the probe liquids. The static CA and CAH as functions of the probe liquids’ surface tensions and dielectric constants selected in the present study are summarized in Figure 1 and Figure 2, respectively. The results demonstrate a good correlation between the liquids properties and the wettability. As observed, all liquids exhibit the general tendency to decrease liquids’ surface tension with reduced static CA, decrease dielectric constant, and reduce CAH. Hence, it can be concluded that the liquids’ polarity has a decisive effect on its wettability. As the correlation variance was large, a further study included a separate wettability analysis of the polar and non-polar liquids. The classification for polar and non-polar liquids was according to their dielectric constant. A dielectric constant higher than 20 was assigned to polar liquids, and a dielectric constant below 20 to non-polar liquids.

**Figure 3:**
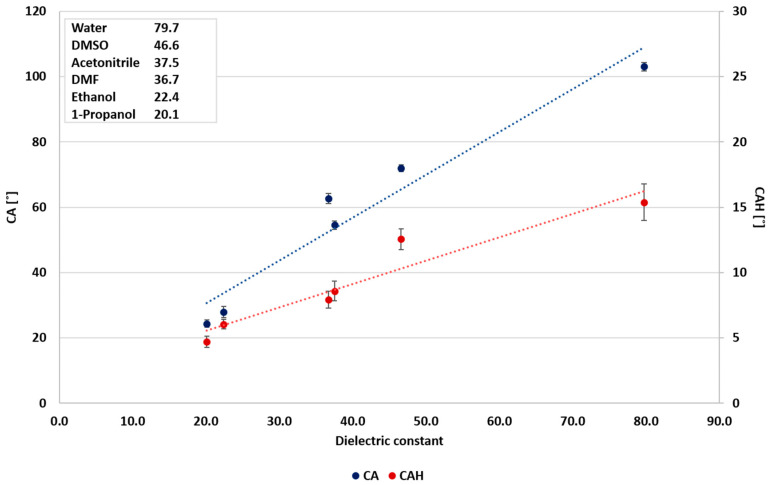
Wettability of polar liquids as a function of the dielectric constant.

**Figure 4:**
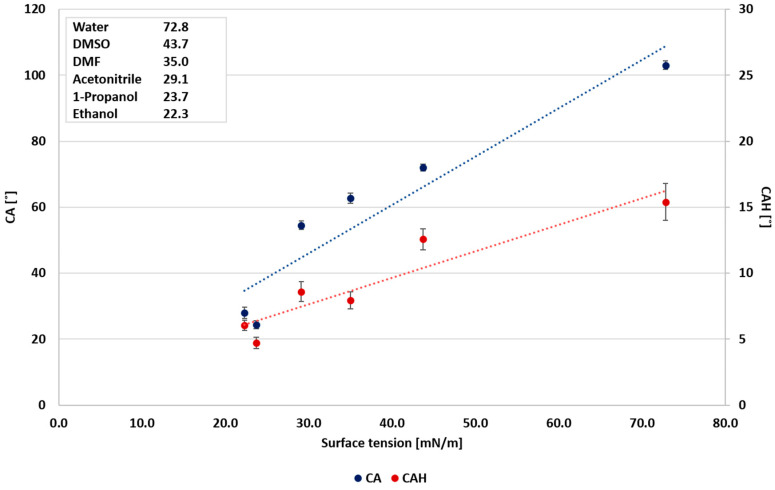
Wettability of polar liquids as a function of surface tension.

**Figure 5:**
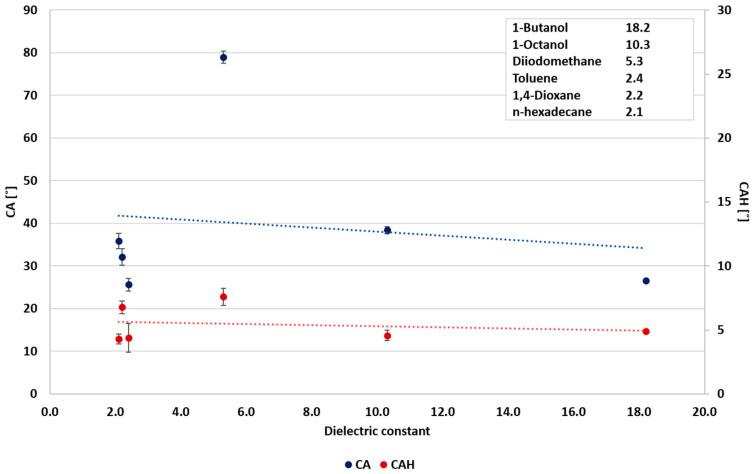
Wettability of non-polar liquids as a function of the dielectric constant.

**Figure 6:**
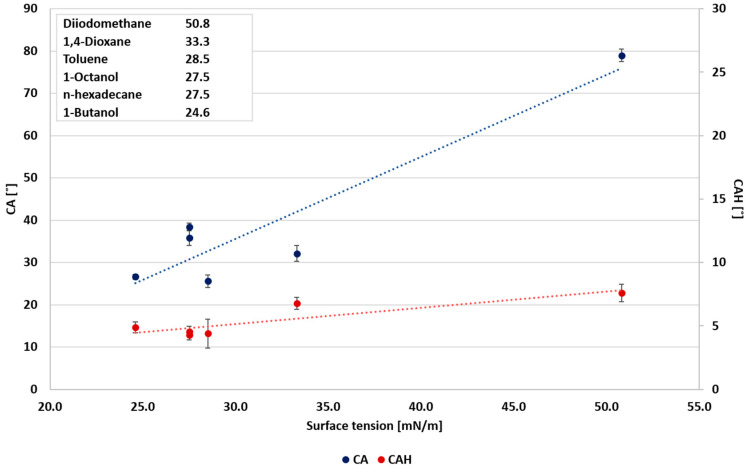
Wettability of non-polar liquids as a function of surface tension.

In the case of polar liquids, it is suggested that CAH is more influenced by the dielectric constant (Figure 3), as pointed out by Urata et al. [8]. However, this study shows that the correlation between the static CA and surface tension and the correlation between static CA and the dielectric constant are similar, as shown in Figure 3 and Figure 4, and as indicated by the correlation coefficients (0.8913 and 0.8942, respectively). Conversely, the static CA and CAH of non-polar liquids displayed a different behavior, where the wettability and the dielectric constant (Figure 5) show no correlation. As shown in Figure 6, a better correlation was obtained between the liquids’ CAH and CA and its surface tension. It should be noticed that n-Hexadecane deviates from the general trend, and it has lower surface tension than 1,4-dioxane and toluene, yet it displays higher static CA.

### 3.2. Sliding Effect of Non-Polar Liquids

**Figure 7:**
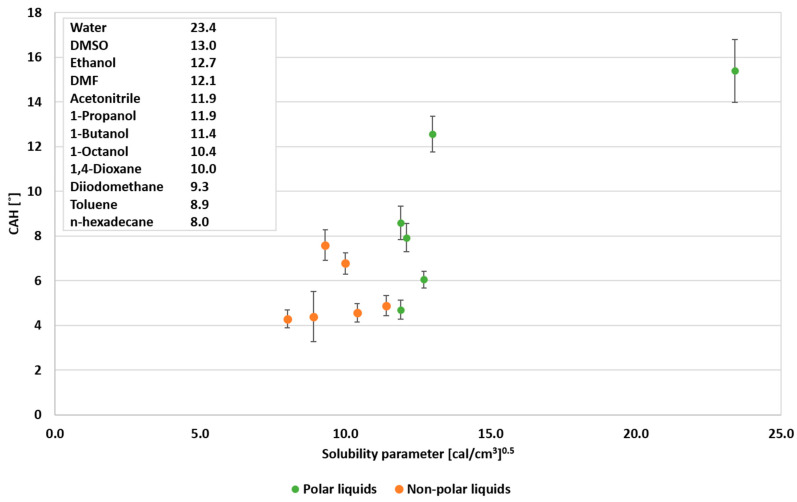
Wettability of probe liquids as a function of the solubility parameter.

**Figure 8:**
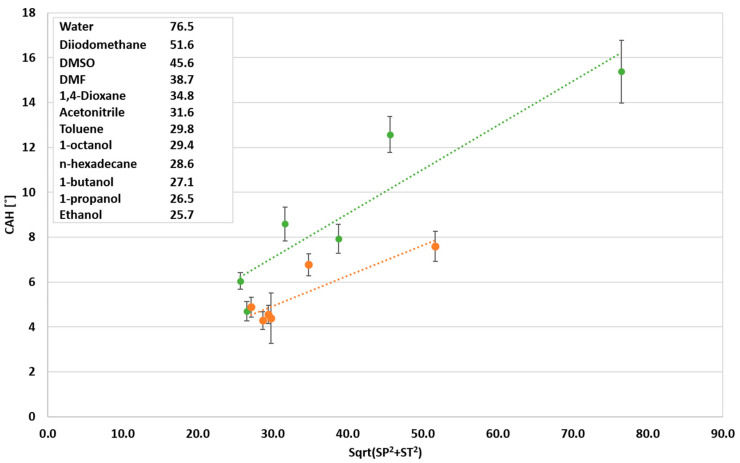
Wettability of probe liquids as a function of the relation between the solubility parameter and surface tension.

As shown in the previous section, the CAH of non-polar liquids is lower than that of polar liquids, indicating the better sliding of the non-polar liquids off the PDMS coating. Thus, in the next stage of the study, the sliding of non-polar liquids and polar liquids on the PDMS treated surface was investigated. Inspired by Cheng et al. [29], the relationship between the dynamic wettability, solubility parameters and the miscibility between the PDMS brushes and the probe liquids have been examined. It is expected that lowering the difference between the solubility parameters will lead to stronger interaction between the probe liquid and the PDMS coating and, consequently, to a decrease in CAH. The wettability of polar and non-polar liquids as a function of solubility parameters is presented in Figure 7. As can be seen, no clear correlation was found between solubility parameters and CAH. Hence, the correlation between CAH and the combination of the solubility parameter and surface tension was analyzed, as presented in Figure 8. The relationship between the CAH and the square root of the combined square power of the solubility parameter and the surface tension (assuming equal contribution of each parameter) for the non-polar liquids exhibits a lower CAH than for the polar liquids. This may be attributed to the influence of the solubility parameter on the CAH that is associated with the actual contact of the liquid with the surface. Hence, in low surface tension, the static CA is lower and the respective contact area is larger. Accordingly, the interaction between the liquid and the surface is enhanced, resulting in the “blended liquid–liquid interface” phenomenon described by Cheng et al. [29]. The latter phenomenon increases the mobility of the PDMS brushes and decreases the CAH. Consequently, according to the present results, it can be proposed that the combination of surface tension and the affinity between the probe liquid and the PDMS (solubility parameter) affects the wettability.

### 3.3. Comprehensive Modeling of CAH

As discussed in the literature and shown in this work, all three liquid properties, namely, the surface tension, dielectric constant and solubility parameter, have a certain effect on the measured CAH. However, the relative contribution of each parameter and their relationships to CAH has not been clarified. In the development of an empirical model that integrates all three parameters, it is important to determine the relationships between the surface tension, γ, and the dielectric constant, ϵ_s_. Papazian et al. [41] described an empirical correlation (Equation (1)) between the surface tension and the dielectric constant of non-polar liquid (with zero dipole moment):

(1) $$\gamma =165*\left(\epsilon_{s}-1\right)/\left(2\epsilon_{s}+1\right)-9.1$$

where $\epsilon_{s}$ is the static dielectric constant and γ is the surface tension (in ergs/cm [2]). Equation (1) cannot relate the surface tensions to the dielectric constants of liquids with finite dipole moment. For the latter case, the Maxwell relation ($n^{2}=\epsilon_{s}\;$(n- index of refraction)) could be used as described by Equation (2), except for strongly hydrogen-bonded liquids.

(2) $$\gamma =286*\left(n^{2}-1\right)/\left(2n^{2}+1\right)-28.6$$

According to Papazian [41], the liquid surface tension, which is a measure of the surface energy per unit area, is the result of the polarization of the liquid molecules in contact with the surface. Hence, the surface tension can be correlated with the dielectric constant. Furthermore, the dispersive component of the solubility parameter, which defines the respective molecule’s dispersive energy, is commonly correlated with other attributes. For example, Koenhen et al. [42] proposed a linear relation between the dispersive component of the solubility parameter and the refractive index, *n*:

(3) $$\delta_{d}=9.55n-5.55$$

Koenhen stated that this correlation (Equation (3)) seemed like the correlation proposed by Papazian between the surface tension and the dielectric constant or the square of the refraction index. Jia and Shi [43] suggested an empirical correlation between the dispersive component of the surface tension and the solubility parameter. They suggested a linear correlation between the dispersive surface tension and the factor $\;\frac{\gamma^{d}}{\delta_{d}^{2/3}}$, as described by Equation (4):

(4) $$\frac{\gamma^{d}}{\delta_{d}^{2/3}}=0.555+0.132\gamma^{d}$$

**Figure 9:**
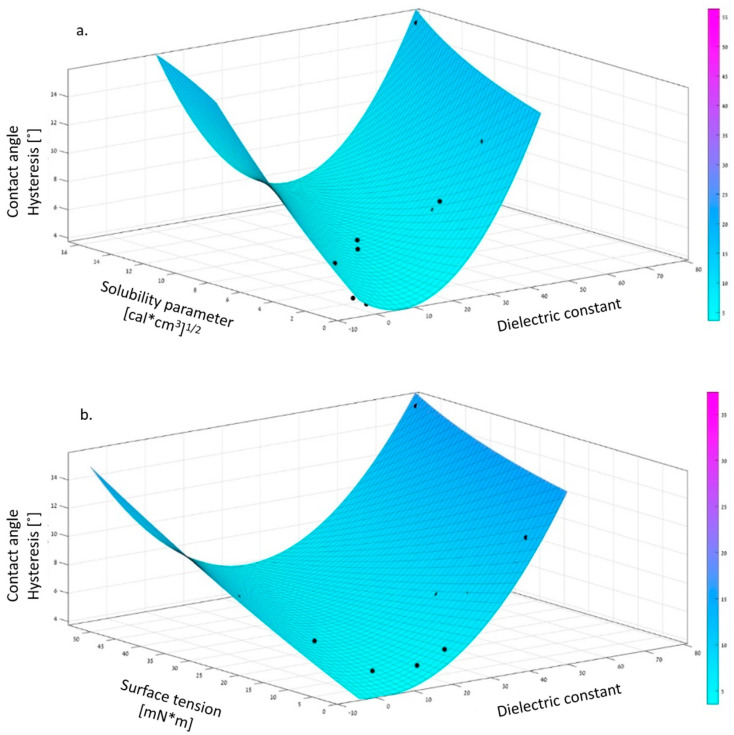
Schematic presentation of the integrated model. (**a**) Contact angle hysteresis (CAH) correlated with the solubility parameter and the dielectric constant. (**b**) CAH correlated with surface tension and the dielectric constant.

Accordingly, an attempt is proposed to integrate all three parameters by a curve fitting approach using a second-order equation (using MatLab software), including interaction terms between the three parameters. It is postulated that the magnitude of the equation’s constants indicates the relative importance of the respective parameter. Twelve probe liquids were used to obtain the empirical correlation, summarized by Equation (5), between the CAH and the three material’s parameters:

(5) $$\mathrm{CAH}=4.2097+0.0130*DC^{2}+0.0051*ST^{2}+0.3389*SP^{2}+0.0074*DC\;*ST-0.0439*ST*SP-0.1294*DC*SP$$

where *DC* is the dielectric constant difference ($∆DC=DC_{liquid}-DC_{PDMS})$, *ST* is the surface tension difference, and *SP* the solubility parameter difference. The correlation coefficient (R [2]) received for the twelve liquids was 0.9768. As can be seen from Equation (5), the solubility parameter has the highest coefficient (0.3389) and the most significant influence on the observed CAH. The lower the difference of the solubility parameters between the coating and the liquid, the lower the CAH. It should be emphasized that the solubility parameter relates to the affinity between the liquid and the surface. A large difference between the solubility parameters of the liquid and the surface will inhibit the chain’s mobility and hence the sliding of the liquid’s drop off the surface. As demonstrated by Equation (5), the dielectric constant difference between the coating and the liquid is of secondary importance and the interfacial tension of tertiary significance for minimizing CAH. It should be noticed that the interaction between the dielectric constant and the solubility parameter, as expressed by its constant (−0.1294), is of significance concerning the reduction of CAH. A graphical scheme of the mathematical model is depicted in Figure 9.

Figure 9 describes the relationship between the solubility parameter differences and the dielectric constant differences and the experimental CAH. As can be observed, a local minimum was obtained at the solubility parameter difference of 2 cal^0.5^/cm^1.5^ and dielectric constant difference of five.

Figure 9 indicates that a local minimum of CAH is obtained at the dielectric constant of eight and interfacial tension of 10 mN/m.

### 3.4. Surface Analysis

**Figure 10:**
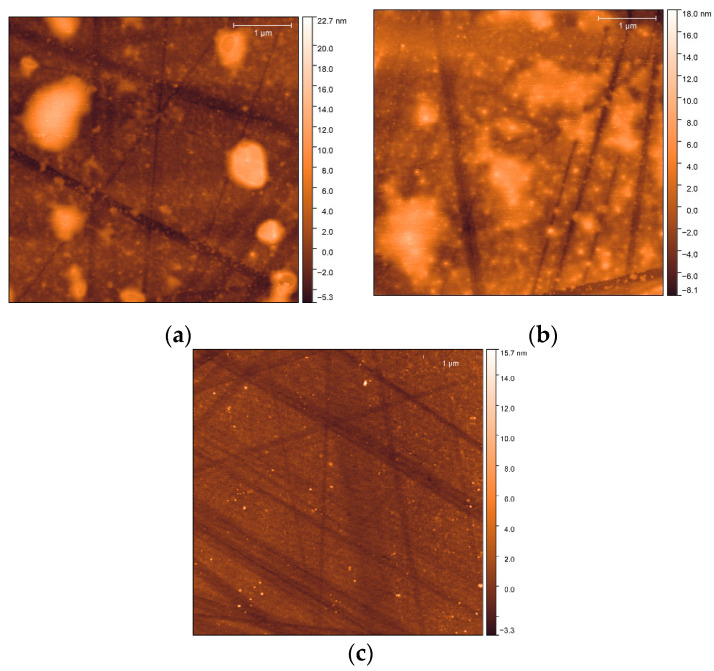
PDMS brush coating in the (**a**) dry state, (**b**) after being submerged in water, and (**c**) after being submerged in n-hexadecane.

The surface smoothness has been analyzed in light of the assumption that PDMS forms a smooth surface by the synthesis proposed by [21,28]. Figure 10a depicts the AFM nanograph of the dry PDMS coating. As evident, the surface is characterized by a random islands 15–20 nm in height. Part of the nanometer roughness and the lines may be attributed to the glass surface morphology following the plasma treatment. However, when submerged in water (polar), the surface becomes more random and the islands heights decrease to 10 nm (Figure 10b). Distinctively, when submerged in n-hexadecane (non-polar), the surface becomes remarkably smoother (Figure 10c) as the islands almost vanish, while the remaining islands are up to 15 nm in height, and their width decreases from 1 µm in the dry state to 100 nm in the wet state.

The AFM numerical data has been used to calculate the average arithmetic value of the profile height deviations from the centerline (Ra). Table 2 presents the calculated values of Ra after submerging the PDMS coating in water and n-hexadecane. After wetting with n-hexadecane, the glass-coated PDMS presents lower values of Ra, indicating a smoother surface. Besides, the PDMS coating presents high standard deviation with water compared to n-hexadecane. These results indicated that the surface has become homogenous after contacting with the good solvent. The AFM analysis indicates that the PDMS coated plasma-treated glass is relatively smooth, having roughness in the nanometer range.

## 4. Conclusions

The static and dynamic wettability of polar and non-polar liquids on a liquid-like PDMS brush coating has been studied as a function of the liquid’s properties. While the liquid’s dielectric constants mostly dictate polar liquid static and dynamic wettability, non-polar liquids show different behavior with no correlation to the liquid’s dielectric constant. Besides the surface tension and dielectric constant, the effect of solubility parameter differences between the PDMS surface and the probe liquids demonstrate that non-polar liquids, having lower solubility parameters than polar liquids, exhibit lower CAH. An empirical mathematical model combining all three parameters provides additional insight and a better understanding of the influence of each material property on CAH. As shown, the solubility parameter is the decisive parameter affecting the CAH, followed by the dielectric constant and only in the third place the surface tension.

It was concluded that omniphobic coatings with low CAH (<15°) could be synthesized based on the combination of the solubility parameter, dielectric constant and surface tension differences between the coating and the various polar and non-polar liquids.

## Figures and Tables

**Table 1:** Physical properties of the probe liquids at 20 °C.

Probe Liquid	Dielectric Constant	Surface Tension (mN/m)	Solubility Parameter (cal·cm^−3^)^0.5^	Viscosity (mPa·S)
Water *^a^*	79.7	72.8	23.4	0.89
Acetonitrile *^a^*	37.5	29.1	11.9	0.38
DMF *^a^*	36.7	35.0	12.1	0.82
Ethanol *^a^*	22.4	22.3	12.7	1.08
Diiodomethane	5.3 *^d^*	50.8 *^c^*	9.3 *^e^*	2.76
Toluene *^a^*	2.4	28.5	8.9	0.59
1,4-Dioxane *^a^*	2.2	33.3 *^c^*	10.0	1.30
n-hexadecane	2.1 *^d^*	27.5 *^c^*	8.0 *^b^*	3.08 *^d^*
1-Propanol *^a^*	20.1	23.7	11.9	1.72
1-Butanol *^a^*	18.2	24.6	11.4	3.0
1-Octanol *^a^*	10.3 *^d^*	27.5	10.4	7.5
DMSO *^a^*	46.6	43.7	13.0	2.0
PDMS *^f^*	2.5	20.8	7.5	−

*^a^* from ref [35], *^b^* from ref [36], *^c^* from ref [37], *^d^* from ref [38], *^e^* from ref [39], *^f^* from ref [40].

**Table 2:** Ra values of PDMS brush coating in the dry state and after contacting water and n-hexadecane.

	Ra (nm)
Dry	2.176±0.953
Water	2.119±0.721
n-hexadecane	0.494±0.056
